# Association of preoperative psychopharmacological treatment and the risk of new chronic opioid use after hip and knee arthroplasty: a Danish registry-based cohort study of 73,033 procedures

**DOI:** 10.2340/17453674.2025.44228

**Published:** 2025-06-28

**Authors:** Simon KORNVIG, Henrik KEHLET, Christoffer C JØRGENSEN, Anders FINK-JENSEN, Poul VIDEBECH, Alma B PEDERSEN, Claus VARNUM

**Affiliations:** 1 Department of Orthopaedic Surgery, Lillebaelt Hospital – Vejle; 2 Department of Regional Health Research, University of Southern Denmark; 3 Section for Surgical Pathophysiology, Copenhagen University Hospital; 4 Centre for Fast-track Hip and Knee Replacement, Rigshospitalet; 5 Department of Anaesthesia and Intensive Care, Hospital of Northern Zealand, Hillerød; 6 Department of Clinical Medicine, University of Copenhagen; 7 Mental Health Center, Copenhagen; 8 Mental Health Center, Glostrup; 9 Department of Clinical Epidemiology, Aarhus University Hospital; 10 Department of Clinical Medicine, Aarhus University, Denmark

## Abstract

**Background and purpose:**

Chronic opioid use is of great concern worldwide. Thus, identification of risk factors for new chronic opioid use (COU) after hip and knee arthroplasty is imperative to target preventive strategies. Depression/anxiety may be risk factors for new COU. However, no studies have investigated whether any or subgroups of preoperative psychopharmacological treatments (PPTs) are risk factors for new COU after hip and knee arthroplasty in a nationwide setting, which was the aim of the present study.

**Methods:**

This population-based cohort study included 40,476 primary hip and 32,557 primary knee arthroplasties from 2015 to 2022 using the Danish Hip/Knee Arthroplasty Registers. Preoperative opioid users were excluded. Dispensing records of psychotropics and opioids were obtained from the Danish National Prescription Registry. Relative risks of new COU were estimated with 95% confidence intervals (CI) using binary regression and adjusted for age, sex, and Charlson Comorbidity Index.

**Results:**

Among hip patients using psychopharmacological treatments (PPTs), 4.6% (202/4,439) had new COU compared with 2.2% (788/36,037) of patients not using PPTs, corresponding to an adjusted relative risk of 1.8 (CI 1.6–2.1). Among total and unicompartmental knee arthroplasties, 9.1% (298/3,261) and 6.4% (59/926) had new COU compared with 4.7 (1,011/21,529) and 2.9% (201/6,841) of patients not using PPTs, corresponding to adjusted relative risks of 1.8 (CI 1.6–2.1) and 2.0 (CI 1.5–2.7), respectively. Analyses of PPT subgroups showed similar results.

**Conclusion:**

Hip and knee arthroplasty patients using PPTs have almost a twofold increased risk of new COU. This emphasizes the need for prevention strategies in these patients.

Chronic opioid use (COU) can induce a vicious cycle of tolerance, dependence, and withdrawal symptoms, causing decreased quality of life and ultimately death [1]. The United States is currently reporting increasing numbers of opioid-related deaths. In 2010, 6.8 per 100,000 Americans died from an opioid-related overdose, whereas 24.7 per 100,000 died in 2021 [2]. Major drivers may include pharmaceutical companies, inadequate regulation, and illegal opioids but also overprescribing [3]. This has led to an increased focus on opioids in other countries, including Denmark [4].

Surgery is an important risk factor for new COU. In Denmark, the incidence of opioid prescriptions in the fourth quarter after hip arthroplasty (THA) among preoperative non-users was 5% from 2013–2018 [5]. Also, an American study found that both minor and major surgeries are risk factors, suggesting that new COU may not be explained entirely by post-surgical pain but also by other risk factors [6]. In THA and knee arthroplasty, several potential risk factors have been identified, e.g., age < 65 years, larger opioid prescriptions on discharge, total knee arthroplasty (TKA), preoperative use, depression, and anxiety [7]. Moreover, a Danish study, performed between 2011 and 2013, has shown that self-reported psychiatric disorder is a risk factor [8]. However, their findings may be influenced by self-report bias and may not reflect current practice as the median length of hospital stay in Denmark decreased from 3 days to 1 day from 2011 to 2020 [9].

The incidence of THAs/TKAs has been projected to increase [10]. Thus, it is paramount to identify high-risk patients to prevent the consequent increase in new COU. No studies have investigated whether any and subgroups of preoperative psychopharmacological treatments (PPTs) are risk factors for new COU after THA, TKA, and unicompartmental knee arthroplasty (UKA) in a nationwide setting, which was the aim of the present study [7].

## Methods

### Study design and setting

We performed a nationwide population-based cohort study using data from the Danish national registries. The unique 10-digit Civil Personal Register (CPR) number issued to every Danish citizen was used to link registry data in this study [11]. All 5.9 million Danish citizens have equal access to tax-financed healthcare. This study is reported in accordance with the REporting of studies Conducted using Observational Routinely-collected health Data (RECORD) Statement [12].

### Study population

We assessed all primary THAs, TKAs, and UKAs due to primary osteoarthritis in Denmark from January 1, 2015 to November 30, 2022 for inclusion using the Danish Hip/Knee Arthroplasty Registries (DHR/DKR), which have a completeness level of more than 95% [13,14]. Patients under 45 years and patients with previous surgery on the same joint were excluded to avoid including patients who may have been misclassified with primary osteoarthritis in the registry. Additional exclusion criteria were simultaneous bilateral procedure, previous contralateral hip or knee arthroplasty within the study period, death within 1 year after surgery, and 1 or more redeemed opioid prescriptions within a half-year before surgery.

### Exposure

Exposures were defined using Anatomical Therapeutic Chemical (ATC) codes, which classify medications by their action and chemical properties. The primary exposure was any preoperative psychopharmacological treatment (PPT) (N05A*, N05B*, N05C*, N06A*, and N06B*), while exposure subgroups were antidepressants (N06A*), serotonin reuptake inhibitors (N06AB* and N06AX16/-21), hypnotics/sedatives (N05C*), anxiolytics (N05B*), and antipsychotics (N05A*).

Since 1995, every redeemed prescription in Denmark has been registered in the Danish National Prescription Registry (NPR). The NPR is considered complete and valid and contains information on CPR number, dispensing date, number of packages, product name, ATC code, package size, tablet/unit strength, prescriber, and pharmacy [15]. However, the intended treatment duration and daily dose are not registered. Thus, the treatment duration for any redeemed PPT prescription was estimated at 99 days for our study population, using a modified version of the waiting time distribution method [16]. This duration was then used to assign exposure status to primary and secondary exposures for each procedure at the time of surgery, as done previously [17].

### Outcome

The outcome, new COU, was defined as at least 2 redeemed opioid prescriptions (ATC codes: N02A*) in the NPR within at least 2 quarters during the last 3 quarters of the first year following surgery. That is, redeemed opioid prescriptions within the first quarter were not included in the outcome definition because opioids can be part of the standard treatment during this time period. Also, the definition was selected to align with a previous study by Edwards et al., which showed that SSRIs, antidepressants, and antipsychotics were risk factors for new COU after hip fracture surgery [18].

### Covariates

All hospitals in Denmark register patients’ primary and secondary discharge diagnoses in the Danish National Patient Registry (DNPR) as International Classification of Diseases 10th edition (ICD-10) codes [19]. This data was used to calculate a Charlson Comorbidity Index (CCI) using primary and secondary diagnoses from a 10-year period before surgery. The 19 Charlson conditions in the DNPR have been validated with a consistently high proportion of confirmed diagnoses [20]. CCI was categorized into 3 groups: no (CCI = 0), low (CCI = 1–2), and high (CCI > 2) comorbidity. Additionally, DHR/DKR provided baseline data on body mass index (BMI), which was categorized as BMI < 25, BMI 25–34.9, and BMI ≥ 35. The DHR included BMI only from the last half of 2016 and onwards.

### Statistics

**Figure 1:**
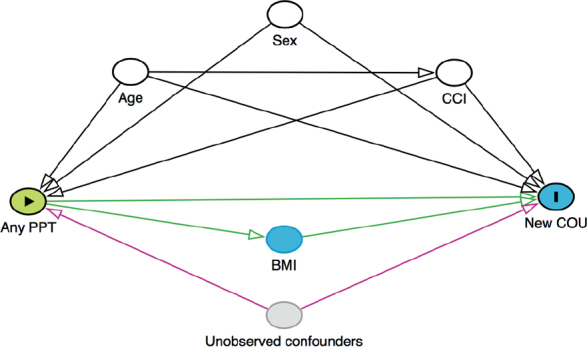
Directed acyclic graph (DAG). PPT: preoperative psychopharmacological treatment; CCI: Charlson Comorbidity Index; COU: chronic opioid use.

Frequencies with percentages or medians with interquartile ranges (IQRs) were used to describe the patient characteristics for each procedure in the study population. Arthroplasty characteristics were compared between patients in any and no PPT using standardized mean differences (SMD). Binary regression was used to calculate relative risks (RRs) of new COU with 95% confidence intervals (CIs). The minimal adjustment set was defined as age, sex, and CCI group based on a directed acyclic graph using DAGitty.net (Figure 1). Importantly, all these covariates were collected before the assignment of exposure status. Time trends in absolute risks of new COU between patients in any and no PPT were calculated and plotted with 95% CIs. All observations in the study were independent, as patients with previous contralateral hip or knee arthroplasty in the study period were excluded. Patients not using PPTs were used as references in all analyses. All analyses were performed using STATA 18.0 BE (StataCorp, College Station, TX, USA).

### Ethics, funding, data sharing, use of AI tools, and disclosures

This study was conducted in compliance with the Declaration of Helsinki and approved by the Record of Data Processing Activities in the Region of Southern Denmark (Journal Number: 22/16325). SK has received funding for salary from the University of Southern Denmark and the Region of Southern Denmark. Lillebaelt Hospital funded the data access at the Danish Health Data Authority. The funders had no role in the planning of the study and were involved in neither the analysis nor the interpretation of the results. Complete disclosure of interest forms according to ICMJE are available on the article page, doi: 10.2340/17453674.2025.44228

## Results

### Study population

**Figure 2:**
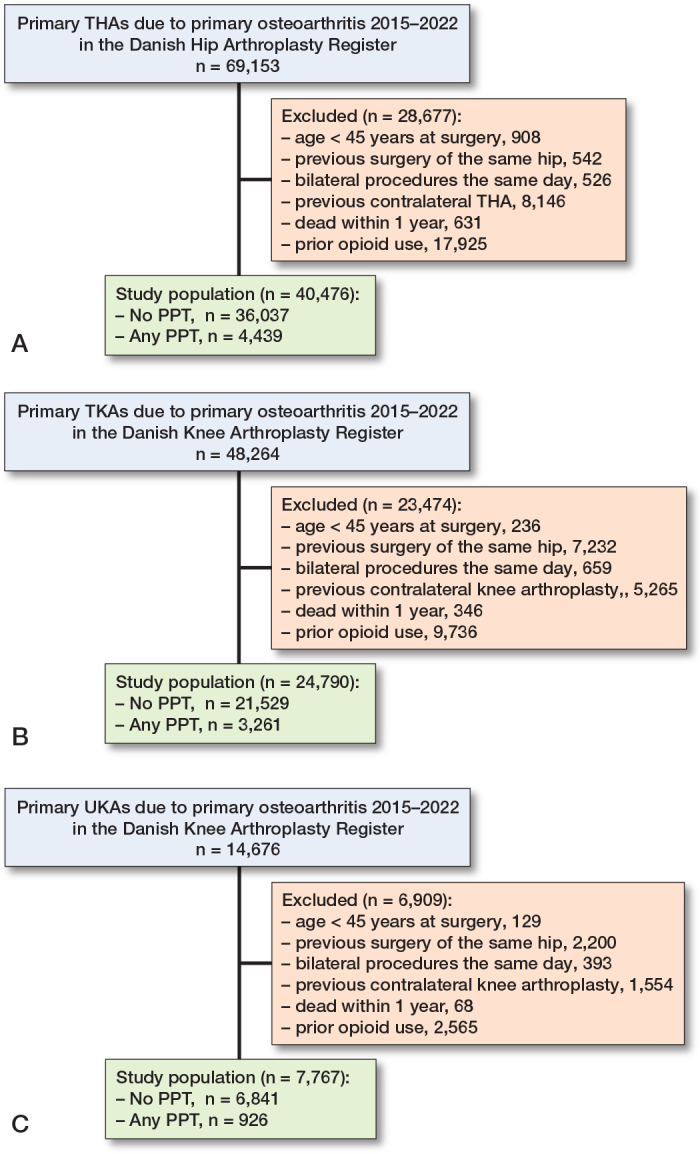
Flowchart of the study populations. A. The total hip arthroplasty (THA) cohort. B. The total knee arthroplasty (TKA) cohort. C. The unicompartmental knee arthroplasty (UKA) cohort. PPT: preoperative psychopharmacological treatment.

This study assessed all 69,153 primary THAs, 48,264 primary TKAs, and 14,676 UKAs in Denmark from January 1, 2015 to November 30, 2022 for inclusion. After exclusion of non-eligible patient, the final study population comprised 40,476 THAs, 24,790 TKAs, and 7,767 UKAs (Figure 2).

Due to the exclusion of patients who had redeemed 1 or more opioid prescriptions within a half-year before surgery, 31%, 28%, and 25% of otherwise eligible THA, TKA, and UKA patients were excluded from the study population, respectively. In the final study population, 11%, 13%, and 12% of THA, TKA, and UKA patients used PPTs, respectively. Patients using PPTs were more likely to be women and had higher CCIs. However, exposed and unexposed patients had similar ages and BMIs. UKAs constituted 24% of all knee arthroplasties (Table 1).

**Table 1:** Arthroplasty characteristics. Values are count (%) unless otherwise specified.

Item	Total hip arthroplasty			Total knee arthroplasty			Unicompartmental knee arthroplasty		
	No PPT	Any PPT	SMD	No PPT	Any PPT	SMD	No PPT	Any PPT	SMD
Number	36,037 (89)	4,439 (11)		21,529 (87)	3,261 (13)		6,841 (88)	926 (12)	
Any PPT	0 (0)	4,439 (100)		0 (0)	3,261 (100)		0 (0)	926 (100)	
Antidepressants		2,661 (60)			2,034 (62)			616 (67)	
SSRIs/SNRIs		1,965 (44)			1,499 (46)			477 (52)	
Hypnotics/sedatives		1,505 (34)			1,092 (33)			300 (32)	
Anxiolytics		611 (14)			443 (14)			105 (11)	
Antipsychotics		427 (10)			385 (12)			78 (8.4)	
Age, median (IQR)	71 (63–76)	72 (65–78)	0.18	72 (65–77)	72 (65–78)	0.04	69 (62–75)	69 (62–75)	0.04
Sex			0.40			0.40			0.39
Female	18,759 (52)	3,148 (71)		12,205 (57)	2,455 (75)		3,420 (50)	639 (69)	
Male	17,278 (48)	1,291 (29)		9,329 (43)	806 (25)		3,421 (50)	287 (31)	
CCI			0.20			0.21			0.16
0	24,787 (69)	2,645 (60)		13,827 (64)	1,796 (55)		4,627 (68)	556 (60)	
1–2	9,008 (25)	1,362 (31)		6,068 (28)	1,063 (33)		1,789 (26)	288 (31)	
> 2	2,242 (6.2)	432 (10)		1,634 (7.6)	402 (12)		425 (6.2)	82 (8.9)	
BMI			0.04			0.09			0.07
< 25	9,599 (27)	1,219 (27)		4,150 (19)	664 (20)		1,432 (21)	199 (21)	
25–35	17,336 (48)	2,046 (46)		14,024 (65)	1,998 (61)		4,615 (67)	605 (65)	
> 35	1,870 (5.2)	257 (5.8)		3,190 (15)	566 (17)		775 (11)	121 (13)	
Missing	7,232 (20)	917 (21)		165 (0.8)	33 (1.0)		19 (0.3)	< 5 (0)	
Year of surgery			0.06			0.05			0.08
2015–2016	8,498 (24)	1,112 (25)		5,111 (24)	831 (25)		1,067 (16)	167 (18)	
2017–2018	8,845 (25)	1,144 (26)		5,076 (24)	765 (23)		1,488 (22)	183 (20)	
2019–2020	9,455 (26)	1,134 (26)		5,864 (27)	885 (27)		1,926 (28)	273 (29)	
2021–2022	9,239 (26)	1,049 (24)		5,478 (25)	780 (24)		2,360 (35)	303 (33)	

PPT: preoperative psychopharmacological treatment; SMD: standardized mean difference; SSRIs: selective serotonin reuptake inhibitors; SNRIs: serotonin-norepinephrine reuptake inhibitors; IQR: interquartile range.

### New chronic opioid use

After THA, 4.6% (CI 4.0–5.2) of patients using PPTs became new COUs compared with 2.2% (CI 2.0–2.3%) of patients not using PPTs. This corresponds to an adjusted RR of new COU of 1.8 (CI 1.6–2.1). After TKA and UKA, 9.1% (CI 8.2–10.2) and 6.4% (CI 4.9–8.1) of patients using PPTs became new COUs, compared with 4.7% (CI 4.4–5.0) and 2.9% (CI 2.6–3.4) of patients not using PPTs, respectively. This corresponds to adjusted RRs of new COU of 1.8 (CI 1.6–2.1) and 2.0 (1.5–2.7), respectively (Table 2).

**Table 2:** Absolute and relative risks of new chronic opioid use after arthroplasty

Item	No PPT	Any PPT	Antidepressants	SSRIs/SNRIs	Hypnotics/sedatives	Anxiolytics	Antipsychotics
Total hip arthroplasty cohort							
Number, n	36,037	4,439	2,661	1,965	1,505	611	427
New COU, n	788	202	125	84	79	38	20
Risk, % (CI)	2.2 (2.0–2.3)	4.6 (4.0–5.2)	4.7 (3.9–5.6)	4.3 (3.4–5.3)	5.2 (4.2–6.5)	6.2 (4.4–8.4)	4.7 (2.9–7.1)
RR (CI)	1 (reference)	2.1 (1.8–2.4)	2.1 (1.8–2.6)	2.0 (1.6–2.4)	2.4 (1.9–3.0)	2.8 (2.1–3.9)	2.1 (1.4–3.3)
aRR (CI)	1 (reference)	1.8 (1.6–2.1)	1.9 (1.6–2.3)	1.8 (1.4–2.2)	2.0 (1.6–2.5)	2.5 (1.8–3.4)	2.0 (1.3–3.1)
Total knee arthroplasty cohort							
Number, n	21,529	3,261	2,034	1,499	1,092	443	385
New COU, n	1,011	298	182	130	113	56	43
Risk, % (CI)	4.7 (4.4–5.0)	9.1 (8.2–10.2)	8.9 (7.7–10,2)	8.7 (7.3–10.1)	10.3 (8.6–12.3)	12.6 (9.7–16.1)	11.2 (8.2–14.7)
RR (CI)	1 (reference)	1.9 (1.7–2.2)	1.9 (1.6–2.2)	1.8 (1.6–2.2)	2.2 (1.8–2.7)	2.7 (2.1–3.5)	2.4 (1.8–3.2)
aRR (CI)	1 (reference)	1.8 (1.6–2.1)	1.8 (1.5–2.0)	1.7 (1.4–2.0)	2.1 (1.7–2.5)	2.5 (1.9–3.2)	2.2 (1.6–2.9)
Unicompartmental knee arthroplasty cohort							
Number, n	6,841	926	616	477	300	105	78
New COU, n	201	59	44	33	20	7	7
Risk, % (CI)	2.9 (2.6–3.4)	6.4 (4.9–8.1)	7.1 (5.2–9.5)	6.9 (4.8–9.6)	6.7 (4.1–10.1)	6.7 (2.7–13.3)	9.0 (3.7–17.6)
RR (CI)	1 (reference)	2.2 (1.6–2.9)	2.4 (1.8–3.3)	2.4 (1.6–3.4)	2.3 (1.5–3.5)	2.3 (1.1–4.7)	3.1 (1.5–6.3)
aRR (CI)	1 (reference)	2.0 (1.5–2.7)	2.3 (1.6–3.1)	2.2 (1.5–3.2)	2.2 (1.4–3.4)	2.2 (1.1–4.6)	2.8 (1.4–5.9)

PPT: preoperative psychopharmacological treatment; SSRIs: selective serotonin reuptake inhibitors; SNRIs: serotonin-norepinephrine reuptake inhibitors; n: frequency; COU: chronic opioid use; RR: crude relative risk; aRR: adjusted relative risk. Adjustments for age, sex, and CCI.

### Exposure subgroups

The most prevalent subgroup of PPTs was antidepressants, with 7% of THA, 8% of TKA, and 8% of UKA patients being users. THA patients using antidepressants had an adjusted RR of new COU of 1.9 (CI 1.6–2.3), while TKA and UKA patients using antidepressants had adjusted RRs of new COU of 1.8 (CI 1.5–2.0) and 2.3 (CI 1.6–3.1), respectively. Similar adjusted RRs were found for serotonin reuptake inhibitors, which constituted the majority of the antidepressants. In both THA and TKA the largest adjusted RRs of 2.5 (CI 1.8–3.4) and 2.5 (CI 1.9–3.2) were found for anxiolytics, respectively. Finally, subgroup analyses of hypnotics/sedatives and antipsychotics yielded adjusted RRs similar to any PPT with larger CIs due to smaller sample sizes (Table 2).

### Time trends

**Figure 3:**
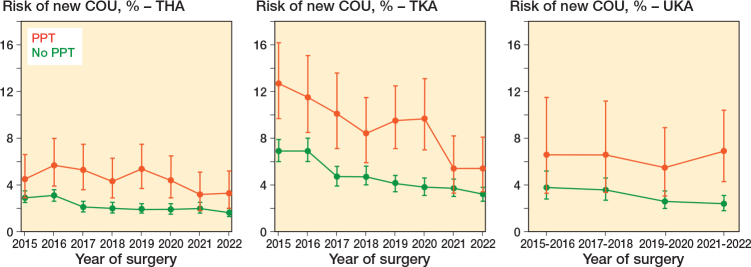
Time trends in absolute risks of new chronic opioid use (COU) for patients in any (red) and no (green) preoperative psychopharmacological treatment stratified by type of surgery.

The absolute risks of new COU after THA and TKA have decreased since 2016 for patients on both any and no PPT. However, the relative risk of new COU between patients on any and no PPT has been on the same level (Table 3, see Appendix). The absolute risk of new COU after UKA has decreased since 2015 for patients with no PPT. This tendency was not seen for UKA patients on any PPT (Figure 3).

**Table 3:** Relative risks of new chronic opioid use by year of surgery

Year	Total hip arthroplasty		Total knee arthroplasty		Unicompartmental knee arthroplasty	
	Crude RR	Adjusted RR	Crude RR	Adjusted RR	Crude RR	Adjusted RR
2015	1.5 (1.0–2.3)	1.3 (0.8–1.9)	1.8 (1.4–2.4)	1.7 (1.3–2.2)	_1.7 (0.9–3.3)_	_1.6 (0.8–3.1)_
2016	1.9 (1.3–2.7)	1.7 (1.2–2.5)	1.7 (1.2–2.3)	1.6 (1.1–2.1)		
2017	2.5 (1.7–3.8)	2.2 (1.5–3.4)	2.1 (1.5–3.1)	2.1 (1.5–3.0)	_1.8 (1.0–3.4)_	_1.7 (1.0–3.2)_
2018	2.1 (1.4–3.3)	2.0 (1.3–3.0)	1.8 (1.2–2.5)	1.5 (1.0–2.2)		
2019	2.8 (1.9–4.1)	2.5 (1.7–3.7)	2.3 (1.7–3.2)	2.3 (1.7–3.2)	_2.1 (1.2–3.6)_	_2.0 (1.1–3.5)_
2020	2.3 (1.5–3.6)	2.0 (1.3–3.1)	2.5 (1.8–3.6)	2.4 (1.6–3.4)		
2021	1.6 (0.9–2.6)	1.3 (0.8–2.3)	1.5 (0.9–2.3)	1.3 (0.8–2.1)	_2.9 (1.8–4.8)_	_2.8 (1.7–4.6)_
2022	2.0 (1.2–3.3)	1.9 (1.1–3.1)	1.7 (1.1–2.7)	1.7 (1.1–2.7)		

RR: relative risk. Adjustments for age, sex, and CCI.

## Discussion

We aimed to investigate the association between PPTs and new COU after hip and knee arthroplasty in a nationwide setting and showed that patients using PPTs have an almost twofold increased risk of new COU.

### Comparison with other studies

A recent narrative review has reported that depression and anxiety are risk factors for prolonged opioid use after hip and knee arthroplasty [7]. Inacio et al. have shown that depression was a risk factor for new COU in 9,122 Australian THA patients with an adjusted odds ratio (OR) of 1.70 (CI 1.20–2.41) defining new COU as 90 days of continuous opioid use or 120 days of non-continuous use [21]. Also, Cancienne et al. reported an OR of prolonged opioid use for depression of 1.32 (CI 1.28–1.36) in 113,337 American TKA patients. Prolonged opioid use was here defined as the filling of an opioid prescription between 3 and 6 months post-surgery [22]. Lastly, Bedard et al. have shown that THA, TKA, and UKA patients with depression/anxiety have a relative risk of COU of 2.2 (CI 2.1–2.4), 2.2 (CI 2.1–2.3), and 2.3 (CI 1.8–2.9) 12 months after surgery, respectively [23-25]. Importantly, both Cancienne et al. and Bedard et al. did not exclude preoperative opioid users from their analyses. Even though these studies used slightly different definitions of COU, their definitions probably produce proportional absolute risks, making their relative outcome measures (e.g., ORs and RRs) comparable to our study. That is, most of these studies have reported relative outcome measures similar to ours. However, the estimate by Cancienne et al. was lower, which is probably explained by different methodologies and populations.

The sub-analysis of anxiolytics in THA and TKA patients yielded the highest RRs of new COU. This is in line with an Australian study of 15,000 patients showing that a history of anxiety increases the risk of COU after initiation [26]. Worryingly, benzodiazepines are associated with a markedly increased risk of death when combined with opioids [27].

Generally, the absolute risks of new COU after THA, TKA, and UKA in our study are decreasing. This is uplifting and a testament that the media attention in 2017 and concurrent regulatory actions by the Danish Health Authority have been fruitful [28]. Since 2018, the number of Danish opioid users has decreased by 12% and the amount of opioids used has decreased by 27%. Additionally, the number of chronic users has decreased by 26%. However, there is still room for improvement as Denmark still has a higher consumption than Sweden [4].

### Strengths

This study used nationwide data with complete data on PPT and new COU. This practically eliminates the problem of selection bias and greatly improves the external and internal validity. All 73,033 eligible primary hip and knee arthroplasties from 2015 to 2022 were included, making this one of the largest studies on PPT and new COU.

### Limitations

Only patients who survived for the first year after surgery could be categorized as new chronic opioid users. Thus, we excluded patients from the analyses who died within the first year, making our results applicable only to first-year survivors. However, the 1-year mortality was low.

We also excluded preoperative opioid users from our study as we were interested in new COU. Future studies may investigate the association between PPT and chronic opioid use after hip and knee arthroplasty in preoperative opioid users. However, the results may be hard to interpret given that preoperative opioid users may use opioids for reasons other than osteoarthritis, e.g., back or shoulder pain, which are not affected by the surgery.

Finally, unmeasured confounders may explain our results even though we adjusted for sex, age, and CCI. To illustrate, Klenø et al. have shown that low education, low wealth, and living alone are associated with analgesic use after THA in Denmark, which would bias our results if low socioeconomic status is also associated with PPT [29]. However, we did not have any data on socioeconomic status. Additionally, serotonin-norepinephrine reuptake inhibitors (SNRIs) and tricyclic antidepressants (TCAs) are also used for pain management, which might confound our results [30].

### Conclusion

We found that hip and knee arthroplasty patients using PPTs had an almost twofold increased risk of new COU. Similar results were found for subgroups of PPTs.

*In perspective,* this study provides the clinician with an easy way to identify patients at increased risk of new COU and subsequently target prevention strategies.

## Notes

SK, HK, CJ, AF, PV, AP, and CV conceptualized and designed the study. SK conducted the data management, the statistical analyses, and drafted the manuscript. SK, HK, CJ, AF, PV AP, and CV participated in the interpretation of the results and the critical revision of the final manuscript.

The authors would like to thank Signe Timm for valuable statistical advice and Open Patient data Explorative Network (OPEN), Odense University Hospital, Region of Southern Denmark for advice regarding data access at the Danish Health Data Authority and the Danish Clinical Quality Program – National Clinical Registries for data extraction from DHR/DKR.

Handling co-editors: Marianne Westberg and Robin Christensen

*Acta* thanks Maaike Gademan and Tina Strømdal Wik for help with peer review of this manuscript.

## References

[1] Mackiewicz M, Brown R E, Price E T, Sargent L. Quality of life in older adults with opioid use disorder: a scoping review. Geriatr Nurs 2022; 46: 118-24. doi: 10.1016/j.gerinurse.2022.05.002. 35679697

[2] Spencer M R, Miniño A M, Warner M. Drug overdose deaths in the United States, 2001–2021. NCHS Data Brief, no 457. Hyattsville, MD: National Center for Health Statistics; 2022. doi: 10.15620/cdc:122556. 36598401

[3] Lancet Regional Health—Americas. Opioid crisis: addiction, overprescription, and insufficient primary prevention. Lancet Reg Health Am 2023; 23: 100557. doi: 10.1016/j.lana.2023.100557. 37497399 PMC10366477

[4] Lægemiddelstatistikregisteret. Forbruget af opioider falder fortsat; 2023. https://sundhedsdatastyrelsen.dk/Media/638676188093096917/Forbrug_opioider_falder_fortsat_2018_2023.pdf

[5] Klenø A N, Sørensen H T, Pedersen A B. Time trends in use of non-steroidal anti-inflammatory drugs and opioids one year after total hip arthroplasty due to osteoarthritis during 1996–2018: a population-based cohort study of 103,209 patients. Osteoarthritis Cartilage 2022; 30(10): 1376-84. doi: 10.1016/j.joca.2022.07.006. 35918050

[6] Brummett C M, Waljee J F, Goesling J, Moser S, Lin P, Englesbe M J, et al. New persistent opioid use after minor and major surgical procedures in US adults. JAMA Surg 2017; 152(6): e170504. doi: 10.1001/jamasurg.2017.0504. Erratum: JAMA Surg 2019; 154(3): 272. doi: 10.1001/jamasurg.2018.5476. 28403427 PMC7050825

[7] VanIderstine C, Johnston E. Risk factors for prolonged opioid use following total hip arthroplasty and total knee arthroplasty: a narrative review of recent literature. Ann Pharmacother 2023; 57(7): 837-46. doi: 10.1177/10600280221133078. 36314233 PMC10291370

[8] Jørgensen C C, Petersen M, Kehlet H, Aasvang E K. Analgesic consumption trajectories in 8975 patients 1 year after fast-track total hip or knee arthroplasty. Eur J Pain 2018 Apr 20. doi: 10.1002/ejp.1232. Online ahead of print. 29676839

[9] Jensen C B, Troelsen A, Foss N B, Nielsen C S, Lindberg-Larsen M, Gromov K. 10-year evolution of day-case hip and knee arthroplasty: a Danish nationwide register study of 166,833 procedures from 2010 to 2020. Acta Orthop 2023; 94: 178-84. doi: 10.2340/17453674.2023.11961. 37074191 PMC10116885

[10] Rupp M, Lau E, Kurtz S M, Alt V. Projections of primary TKA and THA in Germany from 2016 through 2040. Clin Orthop Relat Res 2020; 478(7): 1622-33. doi: 10.1097/CORR.0000000000001214. 32168057 PMC7310374

[11] Schmidt M, Pedersen L, Sørensen H T. The Danish Civil Registration System as a tool in epidemiology. Eur J Epidemiol 2014; 29(8): 541-9. doi: 10.1007/s10654-014-9930-3. 24965263

[12] Benchimol E I, Smeeth L, Guttmann A, Harron K, Moher D, Petersen I, et al.; RECORD Working Committee. The REporting of studies Conducted using Observational Routinely-collected health Data (RECORD) statement. PLoS Med 2015; 12(10): e1001885. doi: 10.1371/journal.pmed.1001885. 26440803 PMC4595218

[13] Danish Hip Arthroplasty Register. National Annual Report for 2022. The Danish Clinical Quality Program – National Clinical Registries (RKKP); 2023.

[14] Danish Knee Arthroplasty Register. National Annual Report for 2022. The Danish Clinical Quality Program – National Clinical Registries (RKKP); 2023.

[15] Pottegård A, Schmidt S A J, Wallach-Kildemoes H, Sørensen H T, Hallas J, Schmidt M. Data resource profile: the Danish National Prescription Registry. Int J Epidemiol 2017; 46(3): 798-798f. doi: 10.1093/ije/dyw213. 27789670 PMC5837522

[16] Pottegård A, Hallas J. Assigning exposure duration to single prescriptions by use of the waiting time distribution. Pharmacoepidemiol Drug Saf 2013; 22(8): 803-9. doi: 10.1002/pds.3459. 23703741

[17] Kornvig S, Kehlet H, Jørgensen C C, Fink-Jensen A, Videbech P, Jakobsen T, et al. Preoperative psychopharmacological treatment is not a risk factor for poorer patient-reported improvements 12 months after hip or knee arthroplasty: a multicenter registry-based cohort study of 7,247 procedures. J Arthroplasty 2025; 40(4): 860-6.e5. doi: 10.1016/j.arth.2024.10.026. 39419411

[18] Edwards N M, Varnum C, Overgaard S, Nikolajsen L, Christiansen C F, Pedersen A B. Risk factors for new chronic opioid use after hip fracture surgery: a Danish nationwide cohort study from 2005 to 2016 using the Danish multidisciplinary hip fracture registry. BMJ Open 2021; 11(3): e039238. doi: 10.1136/bmjopen-2020-039238. PMC7942252 34006019

[19] Schmidt M, Schmidt S A, Sandegaard J L, Ehrenstein V, Pedersen L, Sørensen H T. The Danish National Patient Registry: a review of content, data quality, and research potential. Clin Epidemiol 2015; 7: 449-90. doi: 10.2147/CLEP.S91125. 26604824 PMC4655913

[20] Thygesen S K, Christiansen C F, Christensen S, Lash T L, Sørensen H T. The predictive value of ICD-10 diagnostic coding used to assess Charlson comorbidity index conditions in the population-based Danish National Registry of Patients. BMC Med Res Methodol 2011; 11: 83. doi: 10.1186/1471-2288-11-83. 21619668 PMC3125388

[21] Inacio M C, Hansen C, Pratt N L, Graves S E, Roughead E E. Risk factors for persistent and new chronic opioid use in patients undergoing total hip arthroplasty: a retrospective cohort study. BMJ Open 2016; 6(4): e010664. doi: 10.1136/bmjopen-2015-010664. PMC4853994 27130165

[22] Cancienne J M, Patel K J, Browne J A, Werner B C. Narcotic use and total knee arthroplasty. J Arthroplasty 2018; 33(1): 113-18. doi: 10.1016/j.arth.2017.08.006. 28887020

[23] Bedard N A, Pugely A J, Dowdle S B, Duchman K R, Glass N A, Callaghan J J. Opioid use following total hip arthroplasty: trends and risk factors for prolonged use. J Arthroplasty 2017; 32(12): 3675-9. doi: 10.1016/j.arth.2017.08.010. 28917616

[24] Bedard N A, Pugely A J, Westermann R W, Duchman K R, Glass N A, Callaghan J J. Opioid use after total knee arthroplasty: trends and risk factors for prolonged use. J Arthroplasty 2017; 32(8): 2390-4. doi: 10.1016/j.arth.2017.03.014. 28413136

[25] Bedard N A, DeMik D E, Dowdle S B, Callaghan J J. Trends and risk factors for prolonged opioid use after unicompartmental knee arthroplasty. Bone Joint J 2018; 100-B(1 Supple A): 62-7. doi: 10.1302/0301-620X.100B1.BJJ-2017-0547.R1. 29292342 PMC6424443

[26] Moffat A K, Pratt N L, Kerr M, Kalisch Ellett L M, Roughead E E. Risk of chronic opioid use in older persons with pre-existing anxiety. J Opioid Manag 2019; 16(1): 59-66. doi: 10.5055/jom.2020.0551. 32091618

[27] Park T W, Saitz R, Ganoczy D, Ilgen M A, Bohnert A S. Benzodiazepine prescribing patterns and deaths from drug overdose among US veterans receiving opioid analgesics: case-cohort study. BMJ 2015; 350: h2698. doi: 10.1136/bmj.h2698. 26063215 PMC4462713

[28] Sørensen A M S, Rasmussen L, Ernst M T, Mogensen S H, Laursen M V, Jimenez-Solem E, et al. Use of tramadol and other analgesics following media attention and risk minimization actions from regulators: a Danish nationwide drug utilization study. Eur J Clin Pharmacol 2021; 77(4): 617-24. doi: 10.1007/s00228-020-03016-6. 33112987 PMC7935826

[29] Klenø A N, Stisen M B, Edwards N M, Mechlenburg I, Pedersen A B. Socioeconomic status and use of analgesic drugs before and after primary hip arthroplasty: a population-based cohort study of 103,209 patients during 1996–2018. Acta Orthop 2022; 93: 171-8. doi: 10.2340/17453674.2021.955. 34981126 PMC8815381

[30] Danish Pharmaceutical Information. Medicin.dk. Accessed December 16, 2024. Available from: http://pro.medicin.dk

